# Is Penicillin Plus Gentamicin Synergistic against Clinical Group B *Streptococcus* isolates?: An *In vitro* Study

**DOI:** 10.3389/fmicb.2016.01680

**Published:** 2016-10-21

**Authors:** Corinne Ruppen, Agnese Lupo, Laurent Decosterd, Parham Sendi

**Affiliations:** ^1^Institute for Infectious Diseases, University of BernBern, Switzerland; ^2^Graduate School for Cellular and Biomedical Sciences, University of BernBern, Switzerland; ^3^Division and Laboratory of Clinical Pharmacology, Service of Biomedicine, Department of Laboratories, Centre Hospitalier Universitaire Vaudois (CHUV)Lausanne, Switzerland; ^4^Department of Infectious Diseases, Bern University Hospital, University of BernBern, Switzerland

**Keywords:** Group B *Streptococcus*, *Streptococcus agalactiae*, synergism, gentamicin, aminoglycosides

## Abstract

Group B *Streptococcus* (GBS) is increasingly causing invasive infections in non-pregnant adults. Elderly patients and those with comorbidities are at increased risk. On the basis of previous studies focusing on neonatal infections, penicillin plus gentamicin is recommended for infective endocarditis (IE) and periprosthetic joint infections (PJI) in adults. The purpose of this study was to investigate whether a synergism with penicillin and gentamicin is present in GBS isolates that caused IE and PJI. We used 5 GBS isolates, two clinical strains and three control strains, including one displaying high-level gentamicin resistance (HLGR). The results from the checkerboard and time-kill assays (TKAs) were compared. For TKAs, antibiotic concentrations for penicillin were 0.048 and 0.2 mg/L, and for gentamicin 4 mg/L or 12.5 mg/L. In the checkerboard assay, the median fractional inhibitory concentration indices (FICIs) of all isolates indicated indifference. TKAs for all isolates failed to demonstrate synergism with penicillin 0.048 or 0.2 mg/L, irrespective of gentamicin concentrations used. Rapid killing was seen with penicillin 0.048 mg/L plus either 4 mg/L or 12.5 mg/L gentamicin, from 2 h up to 8 h hours after antibiotic exposure. TKAs with penicillin 0.2 mg/L decreased the starting inoculum below the limit of quantification within 4–6 h, irrespective of the addition of gentamicin. Fast killing was seen with penicillin 0.2 mg/L plus 12.5 mg/L gentamicin within the first 2 h. Our *in vitro* results indicate that the addition of gentamicin to penicillin contributes to faster killing at low penicillin concentrations, but only within the first few hours. Twenty-four hours after antibiotic exposure, PEN alone was bactericidal and synergism was not seen.

## Introduction

*Streptococcus agalactiae* (group B *Streptococcus* [GBS]) is considered a leading cause of morbidity and mortality in neonates and pregnant women. Recommendations for diagnosing maternal GBS colonization and administering intrapartum antimicrobial prophylaxis have led to a significant decrease in these infections (Schrag et al., 2002). Nonetheless, the rate of invasive GBS disease in non-pregnant adults continues to climb (Phares et al., 2008). Elderly persons and those with underlying diseases – two expanding segments of the population – are at increased risk (Skoff et al., 2009). This epidemiological shift is associated with uncertainty in clinical management. Because treatment concepts in adults are not established, those used for neonates – i.e., the combination of β-lactams plus an aminoglycoside (Polin, 2012) – are transferred to adults. For example, some experts advocate this combination therapy for at least the first 2 weeks of treatment for infective endocarditis (IE) (Baddour, 1998; Westling et al., 2007) and periprosthetic joint infection (PJI) (Zimmerli et al., 2004). These recommendations are based on a postulated synergistic effect with penicillin (PEN) and gentamicin (GEN) observed in *in vitro* studies (Cooper et al., 1979; Baker et al., 1981; Swingle et al., 1985). However, adults, especially elderly persons, are more prone to develop side effects caused by aminoglycosides (e.g., nephrotoxicity and ototoxicity) than are neonates (Gonzalez and Spencer, 1998). Here, we evaluated the synergistic effect of PEN and GEN, using contemporary clinical isolates obtained from adults with IE and PJI, and the same antimicrobial products that are administered in clinical practice.

## Materials and Methods

### Bacterial Isolates

Five GBS isolates were used for this study. All isolates were characterized by serotyping (by agglutination [Strep-B-Latex, Statens Serum Institut, Copenhagen, Denmark] and PCR [Imperi et al., 2010]) and multilocus sequence typing (Jones et al., 2003). Two were obtained from patients with PJI and IE (designated as GBS-PJI [BE07-1b] and GBS-IE [BE05-1]). Both were serotype Ib, and sequence type 8. We used three control strains: NEM316 (serotype III, sequence type 23), a strain frequently used in laboratory experiments (Glaser et al., 2002), a colonizing isolate representing a non-virulent strain (designated as GBS-Col [BE12-2], serotype III, sequence type 188), and a previously published high-level GEN-resistant (designated as GBS-HLGR [BSU1203], serotype V, sequence type 7) GBS (Sendi et al., 2016).

### Antibiotics and Concentrations Used

PEN (benzylpenicillin-sodium, Grünenthal Pharma, Mitlödi, Switzerland) and GEN (Hexal AG, Holzkirchen, Germany) were supplied from the clinical pharmacy of the University Hospital (Bern, Switzerland). In time-kill assays (TKAs), PEN concentrations were 0.048 mg/L and 0.2 mg/L, and GEN concentrations were 4 mg/L and 12.5 mg/L. The rationale to use 0.048 mg/L and 0.2 mg/L PEN was based on (i) the setting in previous studies using approximately 1 × MIC (Baker et al., 1981) and (ii) a theoretical extrapolation of PEN concentrations in extravascular compartments. Assuming a penetration proportion of 10–20% (Landersdorfer et al., 2009), and a serum trough level of 3 mg/L (Plaut et al., 1969; Geddes and Gould, 2010), PEN bone concentration is not expected to fall below 0.2 mg/L when intravenous (i.v.) treatment for osteomyelitis or PJI is administered (Zimmerli et al., 2004). The rational to use GEN concentrations of 4 mg/L and 12.5 mg/L is based on GEN peak concentrations found in adults when using i.v., 1 mg/kg or 3 mg/kg (Nicolau et al., 1995). PEN concentrations were diluted from original vials (1 Mio IU), and the concentrations were confirmed via measurements with a UV high-performance liquid chromatography (HPLC) method. GEN concentrations were diluted from original vials (80 mg/2 mL), and the concentrations were confirmed via measurements with a fluorescence polarization immunoassay (COBAS INTEGRA Gentamicin, Roche Diagnostics, Mannheim, Germany). A difference of up to 10% between calculated (i.e., diluted) and measured antimicrobial concentration was allowed in order to proceed with the experiments.

### MIC

Antibiotic susceptibilities for PEN were tested with Etest (Biomérieux, Marcy l’Etoile, France) according to the manufacturer’s protocol, and with microbroth dilution according to protocol (Amsterdam, 2005; Hindler and Tamashiro, 2010). Antibiotic susceptibilities for GEN were tested with microbroth dilution according to protocol (Amsterdam, 2005; Hindler and Tamashiro, 2010). All isolates were tested three or more times.

### Checkerboard Assays

Checkerboard assays were performed as described previously (Pillai et al., 2005; Moody, 2010). In brief, 96-well plates were prepared by serially twofold diluting the first antibiotic (PEN) along the horizontal axis (left [highest concentration; 0.16 mg/L] to right [lowest concertation; 0.0004 mg/L]), and the second antibiotic (GEN) along the vertical axis (top [highest concertation; 250 mg/L] to bottom [lowest concentration; 1.95 mg/L]) in cation-adjusted Mueller Hinton broth (Bacto, Becton, Dickinson and Company, Sparks, MD, USA). Thus, the highest concentration of both antibiotics was in top left well and the lowest of both antibiotics in the bottom right well of the 96-well plate. McFarland 0.5 suspension was prepared and diluted to obtain a final GBS concentration of 3 × 10^5^ to 5 × 10^5^ CFU/mL in each well (Hindler and Tamashiro, 2010). Plates were incubated at 37°C in 5% CO_2_ for 24 h and read out with a microplate reader (Varioskan, Thermo Scientific, Reinach, Switzerland). All assays were repeated at least three times.

### Time-Kill Assays (TKAs)

Time-kill assays were performed according to a previous protocol (Amsterdam, 2005; Moody and Knapp, 2010). Various test conditions were evaluated to determine those that were most stable for GBS (Ruppen and Sendi, 2015). In brief, 1 × 10^5^ to 10^6^ CFU/mL mid-log-phase GBS were incubated in Todd Hewitt broth (Bacto, Becton, Dickinson and Company, Sparks, MD, USA) with either PEN monotherapy, GEN monotherapy, or PEN plus GEN in a total volume of 5 mL at 37°C in 5% CO_2_. Samples of 0.1 mL were obtained at multiple time points up to 24 h after antibiotic exposure, and then plated on Columbia sheep blood agar for colony counting. The lower limit of quantification (LOQ) was defined as 200 CFU/mL and the upper as 3500 CFU/mL [i.e., 20 and 350 CFU, respectively, per plate (Sutton, 2011)]. Assays were repeated multiple times and always performed with triplicates.

### Synergism Assays and Definition

In killing assays, synergy was defined as a ≥100-fold (≥2 log) increase in killing at 24 h (as measured by colony counts [CFU/mL]) with the combination therapy in comparison with the most active single drug (Pillai et al., 2005; Moody and Knapp, 2010). A bactericidal effect was defined as killing of ≥99.9% [i.e., (≥3 log) of the organism within 24 h (Pankey and Sabath, 2004)]. In checkerboard assays, for each strain and for each combination interaction the fractional inhibitory concentration (FIC) of PEN or GEN was calculated (FIC of PEN = MIC of PEN in combination/MIC of PEN alone; FIC of GEN = MIC of GEN in combination/MIC of GEN alone). Then, the FICI (FIC index) was calculated by the summation of both FIC [FICI = FIC of PEN + FIC of GEN (Hindler and Tamashiro, 2010)]. Synergism was defined when FICI resulted in ≤0.5. In the view that this method is a mathematical restatement of an isobologram, “0.5” theoretically reflects the point with one half of the MIC of PEN and one half of the MIC of GEN (Pillai et al., 2005; Moody, 2010). Indifference was defined when the summation of FICI resulted between 0.5< and ≤4, and antagonism when FICI was >4 (Hindler and Tamashiro, 2010)). We did not use the categorization of ‘additive’ because of inherent variability in results derived from the twofold dilution scheme, as described previously (Pillai et al., 2005).

## Results

### MICs, Checkerboard Assays and FICI Results

The results from MICs, checkerboard assays and FICIs for all isolates are shown in **Table 1**. The MIC results are shown as median and range. As expected, all isolates were susceptible to PEN. The MICs were within the same result range when tested with microbroth dilution and Etests. Considering the range of all performed measurements, and a precision error associated with measuring an MIC (i.e., plus or minus one 2-fold dilution) (Turnidge and Paterson, 2007), we determined 0.048 mg/L as concentration to use for these experiments. It was within the range of 1 × MIC for all isolates. The MICs for gentamicin cannot be interpreted (except for the presence of HLGR), because no standard criteria for susceptibility testing are available. The results from checkerboard assays indicated indifference for all isolates, with the lowest FICI for NEM316, ranging from 0.7 to 1. The vast majority of FICI calculations for the other strains resulted in >1 (**Table 1**).

**Table 1:** Antimicrobial susceptibilities (mg/L) and FICIs.

Isolate	MIC (PEN)^MB^	MIC (PEN)^E^	MIC (GEN)^MB^	FICIs^MB^
GBS-PJI	0.03 (0.016–0.04)	0.064 (0.047–0.064)	23.4 (15.6–31.25)	1.4 (0.9–1.5)
GBS-IE	0.04 (0.016–0.04)	0.064 (0.032–0.064)	15.6 (7.8–15.6)	1.4 (0.8–2.3)
NEM316	0.04 (0.032–0.04)	0.064 (0.047–0.064)	15.6 (7.8–15.6)	0.8 (0.7–1)
GBS-Col	0.02 (0.016–0.04)	0.064 (0.047–0.064)	7.8 (7.8–15.6)	2 (1.5–2)
GBS-HLGR	0.04 (0.016–0.04)	0.047 (0.047–0.047)	>1024	2 (2–2)

*All measurements were repeated three or more times. MIC results are presented as median and range of results in parenthesis. E, Etest; MB, microbroth dilution; PEN, penicillin; GEN, gentamicin; FICI, fractional inhibitory concentration index; GBS-PJI, group B *Streptococcus* isolated from a periprosthetic joint infection; GBS-IE, group B *Streptococcus* isolated from infective endocarditis; GBS-Col, group B *Streptococcus* isolated from colonized women; GBS-HLGR, group B *Streptococcus* displaying high level gentamicin resistance.*

### Time-Kill Assays

#### PEN with 0.048 mg/L

**FIGURE 1:**
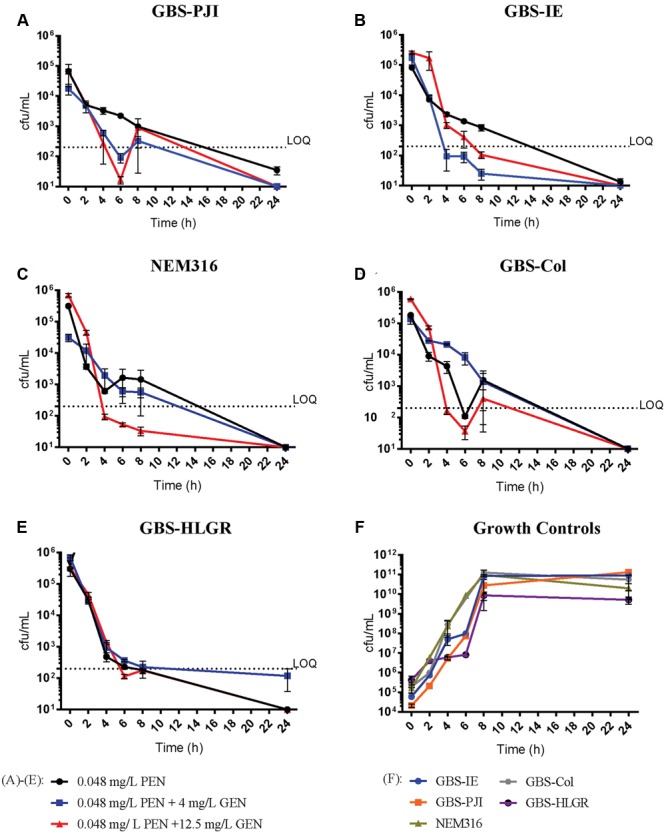
**Time-kill assays with penicillin 0.048 mg/L.** Results are displayed as mean ± standard error of the mean (SEM). PEN 0.048 mg/L monotherapy (●), PEN 0.048 mg/L plus GEN 4 mg/L (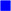) and PEN 0.048 mg/L plus GEN 12.5 mg/L (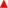). Isolates: GBS-PJI, periprosthetic joint infection **(A)**; GBS-IE, infective endocarditis **(B)**; NEM316, neonatal sepsis **(C)**; GBS-Col, colonization isolate **(D)**; GBS-HLGR, high-level gentamicin resistance **(E)**. Growth controls of all isolates **(F)**. LOQ: lower limit of quantification.

In all isolates, bactericidal killing of GBS at 24 h was observed with PEN monotherapy (**Figure 1**). In isolates obtained from patients with PJI and IE, the killing was better with PEN plus GEN 4 mg/L or PEN plus GEN 12.5 mg/L at 4, 6, and 8 h (**Figures 1A,B**). In NEM316 and the colonizing isolate, killing curves of PEN monotherapy and PEN plus GEN 4 mg/L were similar. Though, the killing was better in NEM316 with PEN plus GEN 12.5 mg/L at 4, 6, and 8 h (**Figure 1C**).

#### PEN with 0.2 mg/L

**FIGURE 2:**
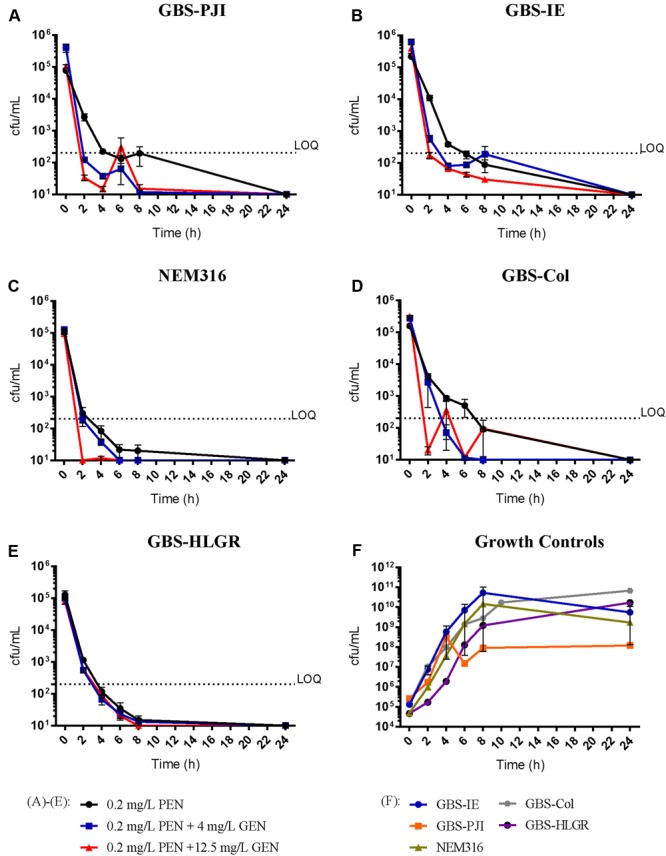
**Time-kill assays with penicillin 0.2 mg/L.** Results are displayed as mean ± standard error of the mean (SEM). PEN 0.2 mg/L monotherapy (●), PEN 0.2 mg/L plus GEN 4 mg/L (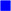), PEN 0.2 mg/L plus GEN 12.5 mg/L (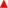). Isolates: GBS-PJI, periprosthetic joint infection **(A)**; GBS-IE, infective endocarditis **(B)**; NEM316, neonatal sepsis **(C)**; GBS-Col, colonization isolate **(D)**; GBS-HLGR, high-level gentamicin resistance **(E)**. Growth controls of all isolates **(F)**. LOQ: lower limit of quantification.

In all isolates but the GBS-Col, killing of GBS decreased the starting inoculum below the LOQ within 4–6 h, irrespective of the addition of GEN (**Figure 2**). In all but the GBS-HLGR isolate, PEN plus 12.5 mg/L GEN showed this killing pattern within 2 h. TKAs with GEN monotherapy showed persistent bacterial growth at 24 h (Supplementary Figure S1).

### Synergism

At 24 h after antibiotic exposure, PEN monotherapy proved to be bactericidal. When synergism was tested with checkerboard assays, none of isolates revealed a FICI value indicating a synergistic effect. Similarly, in TKAs no significant difference was seen in colony counts at 24 h when PEN monotherapy was compared with PEN plus GEN combination therapy. Hence, the lack of synergism correlated in the two laboratory methods.

## Discussion

Rates of invasive GBS disease in non-pregnant adults continue to climb. Hence, clinicians are faced with the challenge of transferring therapeutic knowledge from neonatal sepsis to infections in adults. Other groups previously reported synergism with PEN plus GEN toward GBS (Overturf et al., 1977; Cooper et al., 1979; Baker et al., 1981; Swingle et al., 1985). We used two different methods with isolates obtained from adults and could not confirm these results. The following arguments may explain the differences between the results of previous studies and those provided in the present study.

The definitions and methods used for synergism vary and all have limitations. This again points toward the difficulty to transfer synergy assay results from the laboratory to patient treatment concepts. The two commonly used laboratory methods to evaluate synergism are checkerboard assays and TKAs over a predetermined time interval, as used in the present study. Although the checkerboard method has been reported to have reproducibility problems (Rand et al., 1993), the range of results in our multiple repeated assays was small (**Table 1**). Because the effect of the antibiotic combination in the checkerboard method can be observed at only a single time point, in the TKAs, we used the advantage of measuring the colony counts at various time points up to 24 h after antibiotic exposure. With the last time point, we were able to show a parallel in the results of both methods. In contrast to previous analyses showing a lack of correlation between FICIs and killing curves (Hallander et al., 1982; Bayer and Morrison, 1984; Swingle et al., 1985), our results for both methods were congruent in that they showed no synergistic response.

Some investigators use the first 4–8 h after exposure to an antimicrobial agent or a combination of agents to compare differences in colony counts (Cooper et al., 1979). In our view, this method reflects rapid killing, as shown in our experiments with PEN 0.048 mg/L plus GEN 4 mg/L or 12.5 mg/L (**Figure 1**), or PEN 0.2 m/L plus GEN 12.5 mg/L (**Figure 2**). These results may be helpful to describe potentially beneficial clinical interactions in the very early stage of treatment. Though, they are difficult to standardize (e.g., dynamic of bacterial replication can be variable at different time points). Therefore, differences in colony count measurements at 4 h to 8 h are commonly not used for the definition of synergism (Pillai et al., 2005; Moody and Knapp, 2010). Other means to determine synergy may be more accurate from today’s perspective (e.g., molecular synergy) (McCafferty et al., 1999).

As a consequence of lack of synergism, these results theoretically argue against prolonged combination therapy. The clinical relevance of the laboratory phenomenon “synergism” is difficult to estimate. The bacterial inoculum in time-kill experiments is up to 100,000 times higher than that found in human sepsis (Yagupsky and Nolte, 1990; Puttaswamy et al., 2011). Also, the antibiotic concentrations found in humans treated for bacteremia are significantly higher than the ones used in TKAs. Ten minutes after completion of i.v., administration of 5 million U PEN, the mean serum concentration is 273 mg/L (Plaut et al., 1969; Geddes and Gould, 2010). This corresponds to more than 5,500 times the PEN concentration used in this study. In addition, in severe GBS disease (e.g., IE, PJI), PEN is commonly administered i.v., every 4–6 h (e.g., 18–24 million U/day i.v., in six doses) (Zimmerli et al., 2004; Habib et al., 2009). *In vivo* PEN concentrations decrease in human serum over time. Nonetheless, prior to administration of the next dose, they rarely fall to those levels used in experimental settings. For example, 4 hours after completion of i.v., administration of 5 million IU PEN, the mean serum concentration is 3 mg/L (i.e., >60 times higher than the concentration used in this study) (Plaut et al., 1969; Geddes and Gould, 2010).

In our study, we saw rapid killing of GBS with 0.2 mg/L PEN monotherapy. The killing rate was fastened by 2–4 h when GEN 12.5 mg/L was added. There are no standard concentrations at which antibiotics are tested for synergism. The results with PEN concentrations in the range of 1 × MIC have to be interpreted with caution because of the following reasons. Experiments depend on the precise inoculum (Brook, 1989), the exact determination of MIC, and the antibiotic concentration. In our isolates, PEN MICs were determined by using two different methods and multiple measurements. Moreover, the antibiotic concentrations used were based on calculated dilutions, but confirmed with measurements via fluorescence polarization immunoassay and HPLC.

Various GEN concentrations and products have been used in previous *in vitro* experiments, ranging from 0.5 mg/L to 13 mg/L (Overturf et al., 1977; Cooper et al., 1979; Baker et al., 1981; Kim and Anthony, 1981; Kim, 1987). Since GEN alone in non-toxic systemic concentrations has little or no effect on GBS, the optimal setting for testing synergism in TKAs is unknown. The antibiotic concentrations of combinations used in *in vitro* settings are often not physiological, because PEN is far below achievable serum concentrations, although GEN is within the expected range. On the other hand, higher *in vitro* PEN concentrations diminish the visible GEN effect, and lower GEN concentrations have no effect. The benefit of rapid killing within the first few hours when adding GEN is difficult to interpret for clinical practice, because of the low PEN concentrations used in this study (0.048 mg/L and 0.2 mg/L). Considering the high PEN concentrations and the low inoculum during bacteremia found in humans [e.g., ≤10^2^ cfu/mL; (Yagupsky and Nolte, 1990; Puttaswamy et al., 2011)], we hypothesize no beneficial clinical effect for planktonic bacteria when GEN is added. A clinical trial is required to confirm or reject this hypothesis.

The stability of our results is supported by the small variation seen in multiple assays, comparing TKA results under various conditions (Ruppen and Sendi, 2015) and the use of a HLGR GBS isolate (Sendi et al., 2016).

Another reason for the differences in results found in our study in comparison to previous investigations might be the PEN product. The efficacy of a drug depends largely on the purity of the active ingredient. It is conceivable that the manufacturing process has improved over the decades (van der Beek and Roels, 1984; Penalva et al., 1998). Here, we used PEN products that are administered to patients. However, because we cannot compare older products with the ones used in this study, this statement remains speculative.

We cannot uncritically extrapolate our findings for five isolates to other GBS isolates. Nonetheless, the isolates were obtained from two patients with invasive diseases in which the addition of gentamicin is recommended (IE and PJI). In addition, we used 3 control isolates [two of them previously investigated (Glaser et al., 2002; Sendi et al., 2016)]. The number of isolates used in our study is small. Though, analyzes were performed at multiple time points within the first 8 h for every single isolates. In our view, this information is important in the light that – in clinical practice – PEN is administered every 4–6 h.

## Conclusion

Our study reinvestigated the synergism of PEN plus GEN with two common laboratory methods, clinical isolates in mid-log growth phase and antimicrobial products administered in clinical practice. Synergism according to definition was not observed with either of the methods. In view of the potential nephrotoxicity of aminoglycosides and the increasing elderly population at risk for invasive GBS disease, our findings may have implications for the decision to administer or withhold aminoglycosides.

## Author Contributions

All authors significantly contributed to the generation of this manuscript und fulfilled the criteria for authorship. CR and AL performed the experiments, interpreted the results and co-wrote the manuscript. LD contributed to the acquisition and analyses of the results, co-drafted the work. PS contributed to the conception and design of and interpretation of data for the work, and co-wrote the manuscript.

## Conflict of Interest Statement

The authors declare that the research was conducted in the absence of any commercial or financial relationships that could be construed as a potential conflict of interest.
