# Supplementary material for: Is Penicillin Plus Gentamicin Synergistic against Clinical Group B Streptococcus isolates?: An In vitro Study
Source: Front Microbiol. 2016 Oct 21;7:1680. doi: 10.3389/fmicb.2016.01680 (PMC5073528; doi:10.3389/fmicb.2016.01680)
Supplement: Supplementary file 1 [file Data_Sheet_1.DOCX]

Supplementary Material

Is Penicillin plus Gentamicin Synergistic against Clinical Group B *Streptococcus* isolates?: An *in-vitro* Study.

Corinne Ruppen^1,2^, Agnese Lupo^1^, Laurent Decosterd^3^, Parham Sendi^1,4*^

^1^Institute for Infectious Diseases, University of Bern, Bern, Switzerland

^2^Graduate School for Cellular and Biomedical Sciences, University of Bern, Bern, Switzerland

^3^Division and Laboratory of Clinical Pharmacology, Service of Biomedicine, Department of Laboratories, Lausanne University Hospital (Centre Hospitalier Universitaire Vaudois, CHUV), Lausanne, Switzerland

^4^Department of Infectious Diseases, Bern University Hospital, University of Bern, Bern, Switzerland.

*** Correspondence:**Parham Sendi
[Parham.Sendi@ifik.unibe.ch](mailto:Parham.Sendi@ifik.unibe.ch)

## Supplementary Figures

**Supplementary Figure 1.** Control growth curves with gentamicin monotherapy performed in parallel to time-kill assays with penicillin and penicillin plus gentamicin (Figures 1 and 2).
