# Supplementary figures and images for: Is Penicillin Plus Gentamicin Synergistic against Clinical Group B Streptococcus isolates?: An In vitro Study
Source: Front Microbiol. 2016 Oct 21;7:1680. doi: 10.3389/fmicb.2016.01680 (PMC5073528; doi:10.3389/fmicb.2016.01680)

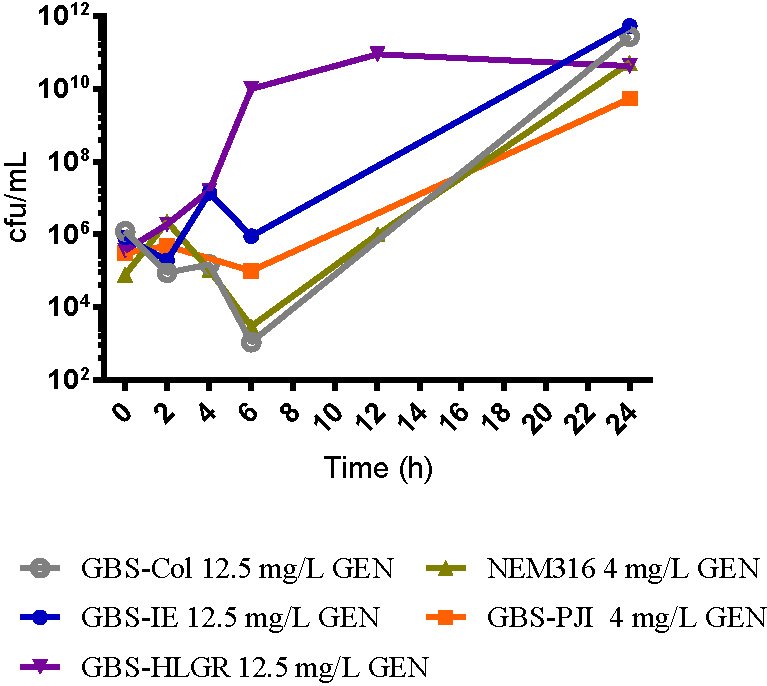

Supplement: Supplementary file 2 [file Image_1.JPEG]
